# Unusually high clarithromycin resistance in *Mycobacterium abscessus* subsp. *abscessus* isolated from human gastric epithelium

**DOI:** 10.3389/fmicb.2023.1193380

**Published:** 2023-08-28

**Authors:** Deepak Chouhan, R. J. Retnakumar, T. Barani Devi, Sanjai Dharmaseelan, Sneha Mary Alexander, Krishnadas Devadas, Santanu Chattopadhyay, Gopinath Balakrish Nair, Madhavan Radhakrishna Pillai

**Affiliations:** ^1^Pathogen Biology Group, Rajiv Gandhi Centre for Biotechnology (RGCB), Thiruvananthapuram, India; ^2^PhD Program in Biotechnology, Manipal Academy of Higher Education (MAHE), Manipal, India; ^3^Department of Gastroenterology, Government Medical College, Thiruvananthapuram, India

**Keywords:** gastric diseases, *Mycobacterium abscessus* subspecies *abscessus*, *Helicobacter pylori*, clarithromcycin resistance, erm (41), antibiotic resisitance

## Abstract

*Mycobacterium abscessus* subsp. *abscessus* is a rapidly growing facultative intracellular pathogen that usually infects human lung and skin epithelium. Recently, we and another group have shown that it also has the potential to colonize human gastric epithelium, but its significance with respect to gastric diseases remains unclear. Although *Helicobacter pylori* still remains the only definite gastric pathogen, recent studies have shown that *M. abscessus* subsp. *abscessus* also has the potential to colonize human gastric epithelium. *M. abscessus* subsp. *abscessus* is known to exhibit multidrug resistance and clarithromycin has been used as the drug of choice. We aimed to determine the clarithromycin resistance profile of 117 (74 rough and 43 smooth) gastric *M. abscessus* subsp. *abscessus* strains and to detect the point mutations in *rrl* and *erm* (41) genes conferring the resistance. Our data showed 79.48% (19 smooth and 74 rough) of *M. abscessus* subsp. *abscessus* strains were resistant to clarithromycin (MIC_90_ ≤ 512 μg/mL), while 20.51% (24 smooth) were susceptible (MIC_90_ ≤ 8 μg/mL). Nucleotide sequence analysis of the *rrl* gene with reference strains of *M. abscessus* subsp. *abscessus* did not show any mutation that is relevant to the clarithromycin resistance. However, analysis of *erm* (41) gene showed that *M. abscessus* subsp. *abscessus* strains, which were susceptible to clarithromycin had C, C, G, and C at their nucleotide positions 28, 159, 238, and 330, respectively, while the resistant strains showed T, T, A, and A at the same positions. Based on antibiogram and sequence analysis data we recommend further studies involving genomic analysis to identify the other genes involved in high clarithromycin resistance in gastric *M. abscessus* subsp. *abscessus* along with the mechanisms involved.

## Introduction

*Mycobacterium abscessus* subspecies *abscessus* (*Mycobacterium abscessus* subsp. *abscessus*) is a non-tuberculous mycobacteria (NTM) and is known for its rapid growth and resistance to multiple drugs. It is known to cause pulmonary infection and skin and soft tissues infections (mostly nosocomial) in humans (Lee et al., 2015). *M. abscessus* subsp. *abscessus* infection is difficult to treat because of its intrinsic resistance to most macrolide and other antibiotics including the classical anti-tuberculous drugs (Nessar et al., 2012; Griffith and Daley, 2022).

Recently, we and another group have isolated *M. abscessus* subsp. *abscessus* from human gastric epithelium. Interestingly, in Trivandrum, Kerala, India, the prevalence of gastric *M. abscessus* subsp. *abscessus* is even higher than the prevalence of *Helicobacter pylori*, a well-known gastric pathogen, which causes gastric cancer and peptic ulcer (Al-Momani et al., 2017; Chouhan et al., 2019). The common treatment regimen for *H. pylori* related gastric diseases is a proton pump inhibitor (e.g., Lansoprazole) and antibiotics. Because of the indiscriminate use of metronidazole to prevent amoebiasis in diarrhoea-endemic places in India and other countries, most *H. pylori* strains are resistant to metronidazole and clarithromycin is mostly the drug of choice against *H. pylori* (Safavi et al., 2016; Gonzales et al., 2019; Shetty et al., 2019). The significance of gastric colonization of *M. abscessus* subsp. *abscessus* with respect to gastric diseases is unknown at present, but the potential of this bacterium to cause diseases should not be neglected. Therefore, the resistance profile of the gastric *M. abscessus* subsp. *abscessus* strains against clarithromycin are worth studying for effective management of gastric diseases.

In 1990s, clarithromycin was the choice of drug to eradicate *M. abscessus* subsp. *abscessus* (Mushatt and Witzig, 1995; Brown-Elliott and Wallace, 2002). Clarithromycin was not effective against *M. abscessus* subsp. *abscessus* with point mutations at A_2058_G, C and A_2059_G, C (*Escherichia coli* numbering) or A_2270_ → G or C and A_2271_ → G or C (*M. abscessus* subsp. *abscessus* numbering) positions in the *rrl* gene that encodes the peptidyltransferese domain of 23S rRNA of bacterial ribosome (Wallace et al., 1996). Apart from mutations in *rrl* gene, another mechanism confers resistance against macrolides in NTM. The *M. abscessus* subsp. *abscessus* strains with functional *erm* (41) gene show an inducible resistance against clarithromycin upon prolonged incubation (14 days), while the *M. abscessus* subsp. *abscessus* strains with non-functional *erm* (41) gene show susceptibility to clarithromycin. In the *erm* (41) gene, a substitution of T-to-C at position 28 (T_28_C), which leads to the alteration of Trp to Arg at the 10^th^amino acid of the peptide, was found to be associated with loss of function and susceptibility to clarithromycin (Nash et al., 2009). The isolates of *M. abscessus* subsp. *massiliense* have not shown any inducible macrolide resistance because of a 397-bp deletion in *erm* (41) gene, which results in a non-functional *erm* (41) gene (Kim et al., 2010).

The aim of this study was to determine the minimum inhibitory concentration (MIC) of the gastric *M. abscessus* subsp. *abscessus* strains against clarithromycin and to understand the genetic basis of the resistance.

## Materials and methods

### Ethics statement

The study was approved by the Institute Human Ethics Committee of Rajiv Gandhi Centre for Biotechnology (Approval Number IHEC/01/2017/18) and by the Human Ethics Committee of Govt. Medical College, Trivandrum (Approval Number IEC.No.05/07/2016/MCT). Patients between the age of 20 and 70 years were recruited for the study and written informed consents were obtained from all patients. Trivandrum is in the southern part of India and is the capital city of Kerala, mostly the part of western ghats with high humidity.

### *Mycobacterium abscessus* subsp. *abscessus* culture and characterization

A total of 117 *M. abscessus* subsp. *abscessus* (rough and smooth) strains isolated from human gastric biopsies were used in this study. The *M. abscessus* subsp. *abscessus* strains were grown on Brain Heart Infusion (BHI) agar plates containing calf serum (7%) and were incubated at 37°C in microaerobic conditions (5% O_2_, 10% CO_2_, 85% N_2_). *M. abscessus* subsp. *abscessus* colonies were identified at the species level based on the growth rate, colony morphology (rough and smooth), and pigmentation as well as 16S rRNA gene sequence analysis. Partial nucleotide sequencing of the *hsp65* gene was used for further confirmation of gastric *M. abscessus* subsp. *abscessus* strains to distinguish them from closely related *M. bolletti, M. chelonae* and *M. massiliense*. Phylogenetic analysis was done by using BioEdit software (version 7.2.6.1).

### Antibiotic susceptibility

*M. abscessus* subsp. *abscessus* strains were tested for clarithromycin (macrolide antibiotic) susceptibility (from 0.125 μg/mL to 512 μg/mL) by agar dilution and broth microdilution assay. BHI plates were prepared using newborn calf serum (7%) and required concentrations of antibiotics, and BHI broth was prepared similarly for the microdilution assay and finally supplemented with antibiotic as per the desired concentration. After 3, 7, and 14 days of incubation with clarithromycin in the microwell, 10 μL liquid culture from each treated well was applied on the BHI plates and were incubated in microaerobic incubator for 3–7 days. To verify the inducible resistance of gastric *M. abscessus* subsp. *abscessus* against clarithromycin, gastric *M. abscessus* subsp. *abscessus* were pre-treated with clarithromycin (0.1 μg/mL) for 3 days and then the MIC was determined. *M. abscessus* subsp. *abscessus* clarithromycin breakpoint (MIC_90_ > 8 μg/mL) was determined according to the Clinical and Laboratory Standards Institute (CLSI) guideline published in 2011. Broth microdilution-based methodology for antimicrobial susceptibility testing of nontuberculous mycobacteria has been considered the gold standard (Woods et al., 2011).

### Bacterial DNA isolation

The bacterial DNA was isolated as previously described (Berg et al., 1997). In brief, the bacterial colonies were harvested in 500 μL PBS and centrifuged at 5,000 rcf for 10 min. The bacterial pellet was resuspended in 200 μL GTE (glucose/tris/EDTA) buffer. The bacterial suspension was then treated with lysozyme (10 mg/mL) at 37°C for 1 h. After enzymatic digestion, bacterial cells were lysed using TES (tris/EDTA/SDS) buffer. Proteinase K (50 μg/mL) and RNase (20 μg/mL) were then added and the tubes were incubated at 55°C for 2 h. The digested bacterial proteins were removed by phenol: chloroform: isoamyl alcohol and then by chloroform: isoamyl alcohol treatments. The bacterial DNA was precipitated using 3 M sodium acetate (pH 5.2) and chilled absolute ethanol. The precipitated DNA was washed with 70% ethanol and the dried pellet was dissolved in 1X TE (tris-EDTA) buffer of pH 8.

### PCR amplification and sequencing of the antimicrobial resistance genes of gastric *Mycobacterium abscessus* strains

Genomic DNA of *M. abscessus* subsp. *abscessus* was used for PCR with primers 16S rRNA V3-V5 F2- (5’-GCC TAC GGG AGG CAG CAG-3′) and V3-V5 R2 (5′-ATT ACC GCG GCT GCT GG-3′) for bacterial 16S rRNA gene (Chouhan et al., 2019); primersHSPF3 (5’-ATC GCC AAG GAG ATC GAG CT-3′) and HSPR4 (5′-AAG GTG CCG CGG ATC TTG TT-3′) for *hsp65* gene sequencing to distinguish *M. abscessus* subsp. *abscessus* from other members of the NTM group (Kim et al., 2005). A total of 46 (31 resistant and 15 susceptible) *M. abscessus* subsp. *abscessus* strains were used to detect antibiotic associated mutations in *M. abscessus* subsp. *abscessus erm* (41) gene, following primers were used:*erm*F (F-GAC CGG GGC CTT CTT CGT GAT-3′) and *erm*R1 (5’-GAC TTC CCC GCA CCG ATT CC-3′) to amplify and *erm* (41)-4 (5′-CCGGCCCGTAGCGTCCAATG-3′) and *erm*F were used for cycle sequencing (Brown-Elliott et al., 2016). Another set of primers ERM1f (5’-CGC CAA CGA GCA GCT CG-3′) and MC823 (5’-GAC TTC CCC GCA CCG ATT CCA C-3′) were used to amplify *erm* (41) gene and to evaluate polymorphism in *erm* (41) gene (Nash et al., 2009; Bastian et al., 2011). To detect mutations in *rrl* gene for acquired resistance in *M. abscessus*, primer (18F 5′-AGT CGG GAC CTA AGG CGA G-3′ and 21R 5’-TTC CCG CTT AGA TGC TTT CAG-3′) were used for amplification and sequencing (Meier et al., 1994). The resulting amplicon of 16S rRNA, *hsp65, erm* (41), and *rrl* gene were purified using Qiaquick PCR purification kit (Qiagen, Hilden, Germany) and were sequenced using BigDye termination v3.1 cycle sequencing kit (Thermo Fisher Scientific, Waltham, Massachusetts, US). Sequencing PCR products were purified by ethanol precipitation and washed with 70% ethanol. The purified products were sequenced using a 3730XL DNA analyser (Thermo Fisher Scientific, Waltham, Massachusetts, US). For the identification of the bacteria, 16S rRNA gene sequence homology analysis was done using BLAST. For phylogenetic classification, multiple *hsp65* gene sequences were assembled and alignment was carried out using ClustalW and phylogenetic tree was constructed as mentioned in Chouhan et al. (2019). To determine the single nucleotide polymorphism (SNP) in *erm* (41) and *rrl* gene of gastric *M. abscessus* strains, the amplified sequence were compared with *M. abscessus* ATCC 19977 genome (GenBank accession number NC_010397.1). We also compared the sequence with *erm* (41) gene of *M. abscessus* strain ATCC19977 (T28 sequevar., GenBank accession number FJ358483.1) and CR5701 (C28 sequevar., GenBank accession number HQ127366.1). For amino acid based protein sequence analysis *M. abscessus* reference strains (GenBank accession number ADM33801.1) were used. All sequences were aligned using ClustalW multiple sequence alignment and were analysed for the mutations at nucleotide as well as amino acid levels using BioEdit software (version 7.2.6.1).

### Nucleotide sequence accession numbers

The *erm* (41) gene sequence of resistant *M. abscessus* subsp. *abscessus* rough (*Mabs* R), resistant *M. abscessus* subsp. *abscessus* smooth (*Mabs* S-A), susceptible *M. abscessus* subsp. *abscessus* smooth (*Mabs* S-B) were submitted to GenBank and accession numbers are MW147115, MW147113, MW147114, respectively. The low molecular weight sequence of *erm* (41) gene was also submitted to GenBank and the accession number is MW142321. Similarly *rrl* gene sequences of resistant *M. abscessus* subsp. *abscessus* rough (*Mabs* R), resistant *M. abscessus* subsp. *abscessus* smooth (*Mabs* S-A), susceptible *M. abscessus* subsp. *abscessus* smooth (*Mabs* S-B) were submitted to GenBank and accession numbers are MW148480, MW148478, and MW148479, respectively.

## Results

### Colony morphologies and clarithromycin resistance patterns of the gastric *Mycobacterium abscessus* strains

The *M. abscessus* subsp. *abscessus* strains isolated from individuals with various gastric diseases have two distinct colony morphologies: smooth and rough. A total of 117 gastric *M. abscessus* subsp. *abscessus* (74 rough and 43 smooth) strains were tested for clarithromycin resistance using agar dilution and broth microdilution based assays. The MIC_90_ for gastric *M. abscessus* subsp. *abscessus* rough morphotypes was ≤256 μg/mL after 14 days of incubation, while *M. abscessus* subsp. *abscessus* rough morphotypes grown in 0.1 μg/mL clarithromycin exhibited an induced increase in MIC_90_ showed MIC_90_ of ≤512 μg/mL after 14 days of treatment (Table 1). Similarly, clarithromycin treatment (uninduced and induced) was carried out for the 24 smooth *M. abscessus* subsp. *abscessus* morphotypes. We observed that all 24 *M. abscessus* subsp. *abscessus* smooth morphotypes had MIC_90_ ≤ 4 μg/mL after 14 days of incubation in uninduced conditions, while in induced conditions, the *M. abscessus* subsp. *abscessus* smooth morphotypes showed MIC_90_ ≤ 8 μg/mL after 14 days of incubation (Table 1). Based on clarithromycin sensitivity pattern we have 3 different types of gastric *M. abscessus* subsp. *abscessus* strains-(a) resistant *M. abscessus* subsp. *abscessus* rough (*Mabs*-R) (b) resistant *M. abscessus* subsp. *abscessus* smooth type A (*Mabs*-S-A) and (c) susceptible *M. abscessus* subsp. *abscessus* smooth type B (*Mabs*-S-B) by considering MIC_90_ ≤ 8 μg/mL as a cut-off in induced as well as uninduced conditions as per the Clinical and Laboratory Standards Institute (CLSI) guideline published in 2011.

**Table 1:** Clarithromycin MIC for gastric *Mycobacterium abscessus* subsp. *abscessus* strains.

*M. abscessus* morphotype (Cla susceptibility)	Clarithromycin MIC_90_ (Uninduced) (μg/mL)	Clarithromycin MIC_90_ (Induced) (μg/mL)	*M. abscessus* (117)
*Mabs* R (resistant)	≤256	≤512	74 (63.24%)
*Mabs* S-A (resistant)	≤256	≤512	19 (16.23%)
*Mabs* S-B (sensitive)	≤4	≤8	24 (20.51%)

*Mabs*-R: *M. abscessus subsp. abscessus* rough, *Mabs*-S-A: *M. abscessus subsp. abscessus* smooth type A, *Mabs*-S-B: *M. abscessus subsp. abscessus* smooth type B and Cla: clarithromycin.

### *Erm* (41) PCR based analysis of *Mycobacterium abscessus* subsp. *abscessus* sensitive and resistant strains

**Figure 1:**
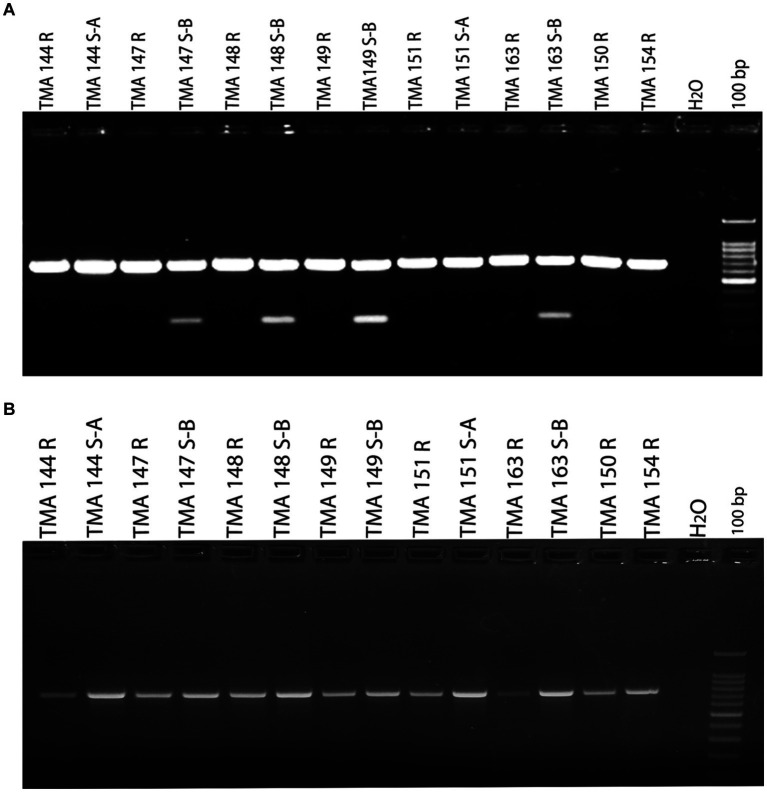
Detection of susceptible and resistant gastric *Mycobacterium abscessus* subsp. *abscessus* isolates using *erm* (41) gene PCR. **(A)** *M. abscessus* subsp. *abscessus* strains (TMA 144 R, 144 S-A, 147 R, 148 R, 149 R, 151 R, 151 S-A, 163 R, 150 R and 154 R) showing single amplicon (670 bp) represent resistant phenotype. *M. abscessus* subsp. *abscessus* strains (TMA 147 S-B,148 S-B, 149 S-B and 163 S-B) showing two amplicon (670 bp and 180 bp) represent susceptible phenotype. **(B)** *erm* (41) gene amplification with another set of primer showing single amplicon for gastric *M. abscessus* subsp. *abscessus* strains. (Mention as ‘100 bp ladder’ in the figure instead of just ‘100 bp).

In order to investigate the molecular basis of clarithromycin resistance in gastric *M. abscessus* subsp. *abscessus* strains, a 670 bp region of the *erm* (41) gene was amplified by PCR. Once *erm* (41) gene was amplified from *M. abscessus* subsp. *abscessus* (rough and smooth) resistant and sensitive strains, the amplified products were visualized on 1.5% of agarose gel. An additional ~180 bp amplicon was observed only for the sensitive strains along with the expected band of 670 bp and the results were consistent for all total 117 strains irrespective of smooth and rough morphotypes (Figure 1A). The additional low molecular weight amplicon (180 bp) has not been reported previously. However, with a different set of primer targeting the *erm* (41) gene, only a single amplicon of 764 bp was observed (Figure 1B).

### Sequence analysis of the *erm (41)* and *rrl* genes

**Figure 2:**
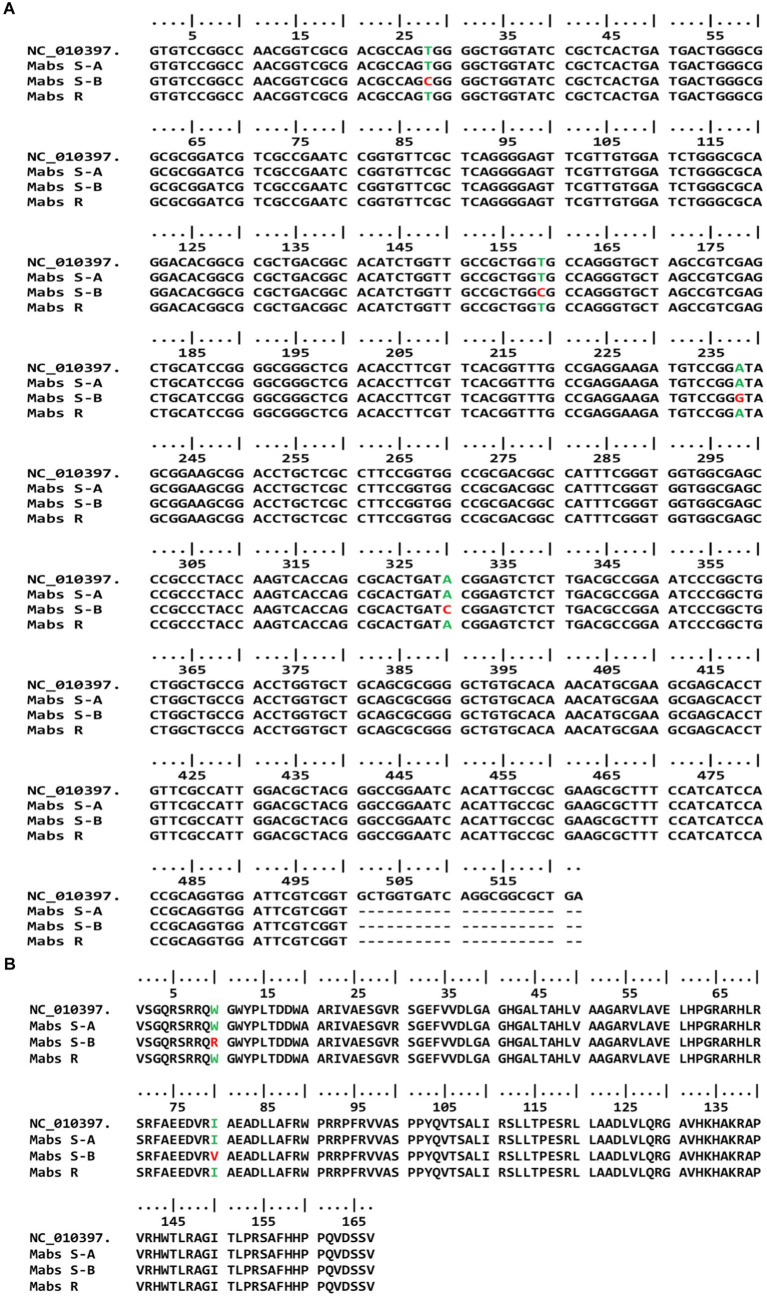
*erm* (41) genotype of susceptible and resistant gastric *M. abscessus* subsp. *abscessus* strains. **(A)** Gene sequence of *erm* (41) showing mutations at 28, 159, 238 and 330 positions in susceptible gastric *M. abscessus* subsp. *abscessus* strains (*Mabs* S-B), while resistant strains (*Mabs* R, *Mabs* S-A) have wild type phenotype. **(B)** Amino acid sequence of Erm (41) protein showing mutations at 10 and 80 amino acid position in susceptible and resistant gastric *M. abscessus* subsp. *abscessus* strains.

As mentioned above, we obtained two amplicons (670 bp and 180 bp) in sensitive and one amplicon (670 bp) in resistant strains. The amplicons were purified and sequenced separately to confirm the association of clarithromycin resistance profiles with single-nucleotide polymorphism (SNP) in the *erm* (41) gene. Earlier reports suggest that SNP at 28 (C to T) nucleotide position is associated with inducible resistance of *M. abscessus* subsp. *abscessus*. We observed all clarithromycin susceptible *M. abscessus* subsp. *abscessus* strains (Pfister et al., 2004) had nucleotide C (GenBank accession number MW147114) and all resistant strains (93) strains had nucleotide T (GenBank accession number MW147113 and MW147115) at the position 28 of *erm* (41) gene (Table 2; Figure 2A). Along with the T_28_C SNP, we also observed nucleotide C at position 159 (T_159_C), nucleotide G at position 238 (A_238_G), and nucleotide C at position 330 (A_330_C) in all susceptible *M. abscessus* strains (GenBank accession number MW147114), but these mutations were absent in all resistant strains of *M. abscessus* (GenBank accession number MW147113 and MW147115), irrespective of smooth and rough morphotypes. The *erm* (41) genes nucleotide sequences were converted to amino acid sequences for both resistant and susceptible *M. abscessus* subsp. *abscessus* strains and was compared with reference strains. We observed Arginine (Arg) and Valine (Val) at position 10 and position 80, respectively only in susceptible strains, while for all resistant strains, Tryptophan (Trp) and Isoleucine (Ile) were observed which is similar to the reference strain (Table 2; Figure 2B). We also amplified *rrl* gene of gastric *M. abscessus* subsp. *abscessus* and sequenced the nucleotides to confirm SNPs in *rrl* gene but we did not observe any SNPs at _2270_A to G or C and _2271_A to G or C (*M. abscessus* numbering) in the *rrl* gene of *M. abscessus* resistant (GenBank accession number MW148480 and MW148478) and susceptible strains (GenBank accession number MW148479) as mentioned in Table 2; Supplementary Figure S1.

**Table 2:** *rrl* and *erm* (41) genotype of susceptible and resistant gastric *M. abscessus* subsp. *abscessus* strains.

*M. abscessus* morphotype (117)	*rrl* gene mutation A_2270_ → G or C A_2271_ → G or C	*erm*(41) mutation T_28_ → C T_159_ → C A_238_ → G A_330_ → C	Erm(41) mutation Trp_10_ → Arg Ile_80_ → Val
*Mabs* R (resistant) (74)	0	0	0
*Mabs* S-A (resistant)	0	0	0
*Mabs* S-B (sensitive)	0	24	24

*Mabs-R*: *M. abscessus subsp. abscessus* rough, *Mabs*-S-A: *M. abscessus subsp. abscessus* smooth type A and *Mabs*-S-B: *M. abscessus subsp. abscessus* smooth type B.

### Sequence analysis of the low molecular weight amplicon of *Mycobacterium abscessus subsp. abscessus*

The low molecular weight amplified product (180 bp) of *oxidoreductase* gene from *M. abscessus* subsp. *abscessus* susceptible strains were sequenced using forward and reverse primers. As shown in Supplementary Figures S3A,B, the chromatograms of Sanger sequencing for both forward and reverse primers showed very distinct peaks.

**Figure 3:**
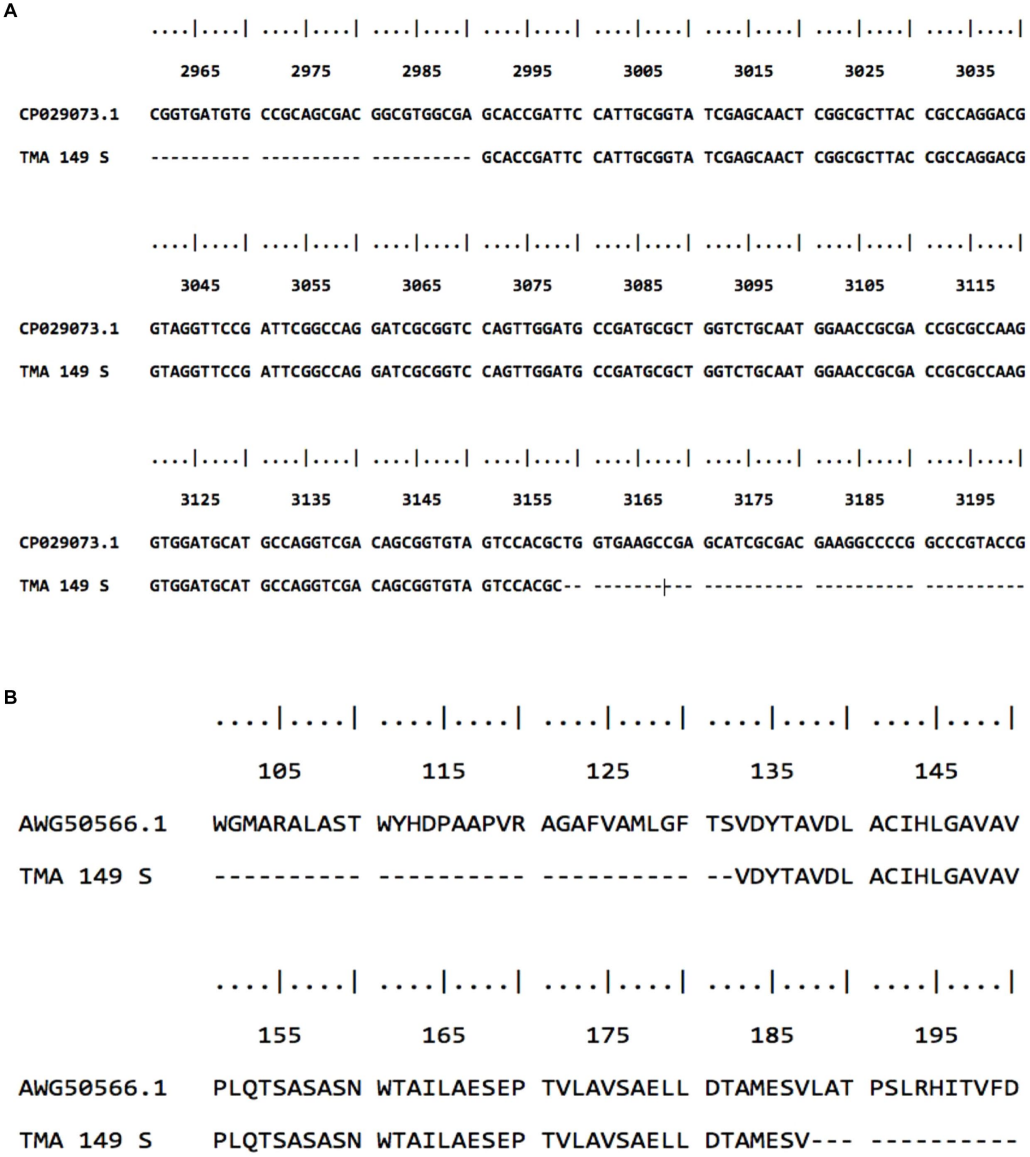
Sequence alignment of *M. abscessus* subsp. *abscessus* strains with low molecular weight amplicon. **(A)** Gene sequence of low molecular weight amplicon shows 100 percent similarity with the oxidoreductase gene of *M. abscesus* subsp. *abscessus* strain G122 chromosome. **(B)** low molecular weight amino acid sequence showed 100 percent similarity with oxidoreductase protein.

Multiple sequence alignment did not show any match of these sequences with the *erm* (41) gene of *M. abscessus* reference strain as well as the gastric *M. abscessus* subsp. *abscessus* strains. To find out the identity of these sequences sequence homology analysis was performed using the BLAST algorithm on the NCBI platform. The BLAST analysis confirmed the identity of these sequences is not *erm* (41) gene but oxidoreductase gene (GenBank: CP029073.1 and CU458896.1) of *M. abscessus* strain G122 and *M. abscessus* ATCC19977. Oxidoreductase of *M. abscessus* contains a total of 3,552 nucleotides, which encode a 1,183 amino acid containing protein. As it was shown in Figure 3, the 180 bp amplicon shows 100 percent similarity with *M. abscessus* G122 strain, starting from G_2991 to C_3158 (GenBank accession number MW142321) which covers 167 bp (Figure 3A). The amino acid sequence starts from valine at the 133 position and the match ends at amino acid valine at the 187 position, which covers a total of 54 amino acids (Figure 3B). With respect to the reference strain *M. abscessus* ATCC19977, the oxidoreductase gene of the gastric *M. abscessus* subsp. *abscessus* showed 4 point mutations at C_3007_ → G, C_3042_ → T, C_3109_ → G and A_3135_ → G (Supplementary Figure S2). Altogether, these results confirmed that the low molecular weight (180 bp) band obtained in the *erm* (41) PCR is amplified from the oxidoreductase gene present in gastric *M. abscessus* subsp. *abscessus* strains.

## Discussion

*M. abscessus* subsp. *abscessus* is a rapidly growing *Mycobacterium* species responsible for pulmonary and soft tissue infections. Although rare, the bacteria also cause disseminated infection, especially in immunocompromised individuals (Lee et al., 2015). *M. abscessus* subsp. *abscessus* is a non-tuberculous mycobacteria (NTM) showing a high level of antibiotic resistance and poses a serious challenge to disease management (Nessar et al., 2012). Various antibiotics which are found to be effective against *M. abscessus* subsp. *abscessus*, like azithromycin, amikacin, meropenem, ciprofloxacin, imipenem, trimethoprim/sulfamethoxazole, and clarithromycin. Among these antibiotics, clarithromycin remains to be the drug of choice to treat *M. abscessus* subsp. *abscessus* related infections (Griffith et al., 2007; Nessar et al., 2012). Recently we reported the presence of *M. abscessus* subsp. *abscessus* in gastric epithelium of patients with various gastric diseases (Chouhan et al., 2019). These gastric diseases are commonly associated with *H. pylori* infection. Recommended treatment option for the eradication of *H. pylori* and the management of related gastric diseases is standard clarithromycin-based triple therapy. In this study, we determined the clarithromycin resistance patterns of gastric *M. abscessus* subsp. *abscessus* strains.

The clarithromycin susceptibility for gastric *M. abscessus* subsp. *abscessus* strains were determined by clarithromycin broth microdilution assay and agar dilution method, where the bacteria was incubated in various concentrations of clarithromycin for 3, 7, and 14 days followed by inspecting bacterial viability. After incubation for 14 days, the growth (viability) pattern of the susceptible and resistant strains were determined. The microdilution-based antibiotic susceptibility test has been recommended by CLSI, because of its reproducibility (Reller et al., 2000; Woods et al., 2011). The clarithromycin susceptibility data obtained in our study of 117 gastric *M. abscessus* subsp. *abscessus* strains demonstrate that the phenotype of clarithromycin susceptibility was fully concordant with *erm* (41) gene SNPs. Our data suggest that of the 117 *M. abscessus* subsp. *abscessus* strains, 93 (79.48%) strains were resistant to clarithromycin, while 24 (20.51%) strains were susceptible to clarithromycin.

The point mutation (C to T) at position 28 of *erm* (41) gene is known to be associated with inducible clarithromycin resistance by *M. abscessus* subsp. *abscessus*. *M. abscessus* subsp. *abscessus* strains showed susceptibility at day 3 of treatment but gradually acquired resistance after 7 days to 14 days of incubation (Nash et al., 2009; Kim et al., 2010; Bastian et al., 2011). Surprisingly we observed there were 19 smooth *M. abscessus* subsp. *abscessus* strains which were resistant to clarithromycin and had wild-type *erm* (41) genotype as like *M. abscessus* subsp. *abscessus* rough morphotype, while susceptible *M. abscessus* subsp. *abscessus* smooth morphotype had SNPs in their *erm* (41) gene. We observed after 14 days of incubation *M. abscessus* subsp. *abscessus* strains type *Mabs* R showed MIC of 256 μg/mL when they were not induced. However, upon induced with clarithromycin, the MIC raised to 512 μg/mL of clarithromycin. *Mabs*S-A strains were susceptible to 256 μg/mL of clarithromycin in uninduced conditions and MIC was 512 μg/mL in induced conditions. On the other hand, *Mabs*S-B showed MIC of 8 μg/mL after induction, when compared to 4 μg/mL of clarithromycin in uninduced conditions. We observed resistant phenotype with wild type *erm* (41) gene with T, T, A, and A nucleotide base at the 28, 159, 238, and 330 positions, respectively but strains with C, C, G, and C at the 28, 159, 238, and 330 nucleotide positions respectively, exhibited susceptible phenotype (Tables 1, 2). Acquired and inducible resistance for clarithromycin in *M. abscessus* has already been reported. Acquired resistance for clarithromycin has been associated with point mutations at A_2270_ → G or C and A_2271_ → G or C (*M. abscessus* numbering system) of *rrl* gene (Pfister et al., 2004; Lipworth et al., 2018). To our surprise, we did not observe any mutations in gastric *M. abscessus* subsp. *abscessus* strains at position A_2270_ → G or C and A_2271_ → G or C (*M. abscessus* numbering system) of *rrl* gene. In conclusion, no association was observed between *rrl* gene and clarithromycin MIC for the resistant and sensitive phenotype of gastric *M. abscessus* subsp. *abscessus* strains.

Mutation from isoleucine (Ile) to valine (Val) at the 80^th^ position has been associated with macrolide drug resistance in *M. abscessus* strains isolated from Korea. Nash et al. has shown that Erm41 protein with Trp10 was associated with resistance while Arg10 was associated with susceptible phenotype, as the protein harbouring Arg10 was non-functional (Nash et al., 2009; Lee et al., 2014). Our amino acid sequence analysis for Erm (41) protein suggests that all susceptible strains of *M. abscessus* subsp. *abscessus* had Arginine (Arg) amino acid at 10^th^ position, while Tryptophan (Trp) was present in resistant strains of *M. abscessus* subsp. *abscessus*. We also observed mutation at position 80, where Valine (Val) was replaced by Isoleucine (Ile) in resistant strains of gastric *M. abscessus* subsp. *abscessus*. Our *erm* (41) gene analysis for gastric *M. abscessus* subsp. *abscessus* strains correlated with susceptibility pattern of *M. abscessus* subsp. *abscessus* strains irrespective of smooth and rough morphotypes. The sequence of low molecular weight (180 bp) amplicon did not show any homology with *erm* (41) gene of *M. abscessus* strains but BLAST analysis identified that 180 bp amplicon belongs to oxidoreductase gene of *M. abscessus* strain G122 with 100 percent match. On the other hand, when compared with *M. abscessus* ATCC19977 strain, the 180 bp amplicon sequence displayed 4 point mutations, C_3007_ → G, C_3042_ → T, C_3109_ → G and A_3135_ → G in oxidoreductase gene (Supplementary Figure S2). Most of the antibiotics also exert their bactericidal effect by generating reactive oxygen species (ROS) or targeting bacterial redox systems. The bacterial oxidoreductase gene has been linked to antibiotic resistance through neutralizing toxic molecules, detoxification of antibiotics, and repair damage caused by antibiotics. Recent studies have revealed that *M. abscessus* subsp. *abscessus* induces efflux pump encoding genes in response to antibiotic stress specifically antibiotic targeting ribosome (Egorov et al., 2018; Mudde et al., 2022; Schildkraut et al., 2022). A mutation in the oxidoreductase gene leading to a non-functional oxidoreductase enzyme may exert a detrimental effect on bacteria during antibiotic treatment. In this study, we observed a possible link between the oxidoreductase gene of *M. abscessus* subsp. *abscessus* in clarithromycin resistance. Further study involving whole genome sequencing of gastric *M. abscessus* subsp. *abscessus* strains are needed to understand the antibiotic resistance gene pool present in the strains along with genotypes and the nature of resistance (acquired or induced). To conclude, we determined the clarithromycin resistance profile of the gastric *M. abscessus* subsp. *abscessus* strains and studied the genetic basis of clarithromycin resistance. We have also demonstrated induced clarithromycin resistance in the isolated gastric *M. abscessus* subsp. *abscessus* strains. Our finding also suggests that the C to T mutation at the 28^th^ nucleotide position of the *erm* (41) gene has an important role in conferring clarithromycin resistance and *erm* (41) gene sequence analysis can light on the mechanism of clarithromycin resistance in of gastric *M. abscessus* subsp. *abscessus*. The results of our study will be helpful while designing efficient strategies to combat multi-drug resistant strains of *M. abscussus* subsp. *abscessus* as well as for the management of associated gastric diseases.

## Data availability statement

The datasets presented in this study can be found in online repositories. The names of the repository/repositories and accession number (s) can be found in the article/Supplementary material.

## Ethics statement

The studies involving humans were approved by Institute Human Ethics Committee of Rajiv Gandhi Centre for Biotechnology (Approval Number IHEC/01/2017/18) and by the Human Ethics Committee of Govt. Medical College, Trivandrum (Approval Number IEC.No.05/07/2016/MCT). The studies were conducted in accordance with the local legislation and institutional requirements. The participants provided their written informed consent to participate in this study.

## Author contributions

MP conceptualized the idea. DC, TD, KD, SD, and RR performed the experiments. DC, SC, KD, GN, and MP analyzed the data. DC, SC, RR, KD, GN, and MP wrote the manuscript. All authors contributed to the article and approved the submitted version.

## Funding

This study was supported by Rajiv Gandhi Centre for Biotechnology (RGCB), an autonomous institute under Department of Biotechnology (DBT) and grant from Department of Biotechnology, Government of India to MRP and Department of Science and Technology (ECR/2016/000171) to SC.

## Conflict of interest

The authors declare that the research was conducted in the absence of any commercial or financial relationships that could be construed as a potential conflict of interest.

## Publisher’s note

All claims expressed in this article are solely those of the authors and do not necessarily represent those of their affiliated organizations, or those of the publisher, the editors and the reviewers. Any product that may be evaluated in this article, or claim that may be made by its manufacturer, is not guaranteed or endorsed by the publisher.
