# Supplementary material for: Unusually high clarithromycin resistance in Mycobacterium abscessus subsp. abscessus isolated from human gastric epithelium
Source: Front Microbiol. 2023 Aug 28;14:1193380. doi: 10.3389/fmicb.2023.1193380 (PMC10494244; doi:10.3389/fmicb.2023.1193380)
Supplement: Supplementary file 1 [file Data_Sheet_1.pdf]

Supplementary Figure

```
              ....|....| ....|....| ....|....| ....|....| ....|....| ....|....| ....|....| ....|....| ....|....|
                   1985       1995       2005       2015       2025       2035       2045       2055       2065
NC_010397.    CGACTGTTTA CTAAAAACAC AGGTCCGTGC GAAGTCGCAA GACGATGTAT ACGGACTGAC GCCTGCCCGG TGCTGGAAGG TTAAGAGGAC
Mabs S-A      CGACTGTTTA CTAAAAACAC AGGTCCGTGC GAAGTCGCAA GACGATGTAT ACGGACTGAC GCCTGCCCGG TGCTGGAAGG TTAAGAGGAC
MabsS-B       CGACTGTTTA CTAAAAACAC AGGTCCGTGC GAAGTCGCAA GACGATGTAT ACGGACTGAC GCCTGCCCGG TGCTGGAAGG TTAAGAGGAC
Mabs R        CGACTGTTTA CTAAAAACAC AGGTCCGTGC GAAGTCGCAA GACGATGTAT ACGGACTGAC GCCTGCCCGG TGCTGGAAGG TTAAGAGGAC

              ....|....| ....|....| ....|....| ....|....| ....|....| ....|....| ....|....| ....|....| ....|....|
                   2075       2085       2095       2105       2115       2125       2135       2145       2155
NC_010397.    CCGTTAACCC TTGGGTGAAG CGGAGAATTT AAGCCCCAGT AAACGGCGGT GGTAACTATA ACCATCCTAA GGTAGCGAAA TTCCTTGTCG
Mabs S-A      CCGTTAACCC TTGGGTGAAG CGGAGAATTT AAGCCCCAGT AAACGGCGGT GGTAACTATA ACCATCCTAA GGTAGCGAAA TTCCTTGTCG
MabsS-B       CCGTTAACCC TTGGGTGAAG CGGAGAATTT AAGCCCCAGT AAACGGCGGT GGTAACTATA ACCATCCTAA GGTAGCGAAA TTCCTTGTCG
Mabs R        CCGTTAACCC TTGGGTGAAG CGGAGAATTT AAGCCCCAGT AAACGGCGGT GGTAACTATA ACCATCCTAA GGTAGCGAAA TTCCTTGTCG

              ....|....| ....|....| ....|....| ....|....| ....|....| ....|....| ....|....| ....|....| ....|....|
                   2165       2175       2185       2195       2205       2215       2225       2235       2245
NC_010397.    GGTAAGTTCC GACCTGCACG AATGGCGTAA CGACTTCTCA ACTGTCTCAA CCATAGACTC GGCGAAATTG CACTACGAGT AAAGATGCTC
Mabs S-A      GGTAAGTTCC GACCTGCACG AATGGCGTAA CGACTTCTCA ACTGTCTCAA CCATAGACTC GGCGAAATTG CACTACGAGT AAAGATGCTC
MabsS-B       GGTAAGTTCC GACCTGCACG AATGGCGTAA CGACTTCTCA ACTGTCTCAA CCATAGACTC GGCGAAATTG CACTACGAGT AAAGATGCTC
Mabs R        GGTAAGTTCC GACCTGCACG AATGGCGTAA CGACTTCTCA ACTGTCTCAA CCATAGACTC GGCGAAATTG CACTACGAGT AAAGATGCTC

              ....|....| ....|....| ....|....| ....|....| ....|....| ....|....| ....|....| ....|....| ....|....|
                   2255       2265       2275       2285       2295       2305       2315       2325       2335
NC_010397.    GTTACGCGCG GCAGGACGAA AAGACCCCGG GACCTTCACT ATAGCTTGGT ATTGGCGTTT GGTTCGGTTT GTGTAGGATA GGTGGGAGAC
Mabs S-A      GTTACGCGCG GCAGGACGAA AAGACCCCGG GACCTTCACT ATAGCTTGGT ATTGGCGTTT GGTTCGGTTT GTGTAGGATA GGTGGGAGAC
MabsS-B       GTTACGCGCG GCAGGACGAA AAGACCCCGG GACCTTCACT ATAGCTTGGT ATTGGCGTTT GGTTCGGTTT GTGTAGGATA GGTGGGAGAC
Mabs R        GTTACGCGCG GCAGGACGAA AAGACCCCGG GACCTTCACT ATAGCTTGGT ATTGGCGTTT GGTTCGGTTT GTGTAGGATA GGTGGGAGAC
```

Figure S1. *rrl* genotype of susceptible and resistant gastric *M. abscessus* subsp. *abscessus* strains showing wild type *rrl* genetic sequence once compared with reference *M. abscessus* subsp. *abscessus* strains.

```
              ....|....| ....|....| ....|....| ....|....| ....|....| ....|....| ....|....| ....|....|
                   2965       2975       2985       2995       3005       3015       3025       3035
CU458896.1    CGGTGATGTG CCGCAGCGAC GGCGTGGCGA GCACCGATTC CATTGCCGTA TCGAGCAACT CGGCGCTTAC CGCCAGGACG
TMA 149 S     ---------- ---------- ---------- GCACCGATTC CATTGCGGTA TCGAGCAACT CGGCGCTTAC CGCCAGGACG

              ....|....| ....|....| ....|....| ....|....| ....|....| ....|....| ....|....| ....|....|
                   3045       3055       3065       3075       3085       3095       3105       3115
CU458896.1    GCAGGTTCCG ATTCGGCCAG GATCGCGGTC CAGTTGGATG CCGATGCGCT GGTCTGCAAT GGAACCGCCA CCGCGCCAAG
TMA 149 S     GTAGGTTCCG ATTCGGCCAG GATCGCGGTC CAGTTGGATG CCGATGCGCT GGTCTGCAAT GGAACCGCGA CCGCGCCAAG

              ....|....| ....|....| ....|....| ....|....| ....|....| ....|....| ....|....| ....|....|
                   3125       3135       3145       3155       3165       3175       3185       3195
CU458896.1    GTGGATGCAT GCCAAGTCGA CAGCGGTGTA GTCCACGCTG GTGAAGCCGA GCATCGCGAC GAAGTCCCCG GCCCGTACCG
TMA 149 S     GTGGATGCAT GCCAGGTCGA CAGCGGTGTA GTCCACGC-- ---------- ---------- ---------- ----------
```

Figure S2. *M. abscessus* ATCC19977 showed similarity for low molecular weight sequence but with mutations at C_{3007}→G, C_{3042}→T, C_{3109}→G and A_{3135}→G.

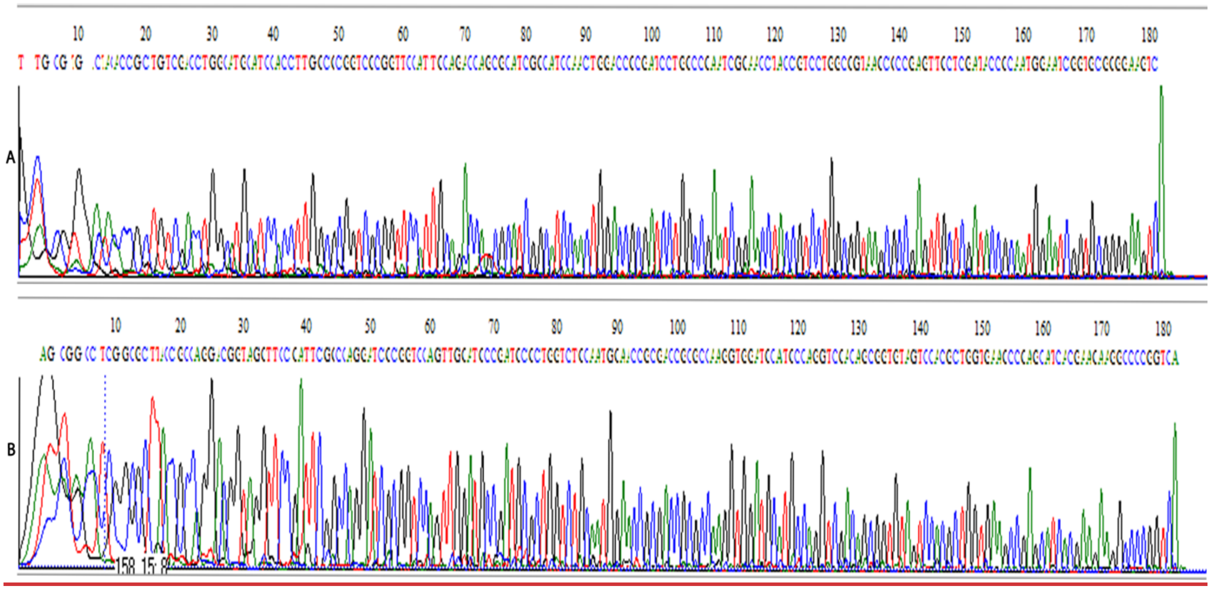

Figure S3. Sequencing chromatogram. A. chromatogram of forward primer derived nucleotide sequence of gastric *M. abscessus* subsp. *abscessus*. **B** chromatogram of reverse primer derived nucleotide sequence of gastric *M. abscessus* subsp. *abscessus*.
